# The dual-target approach in viral HIV-1 viremia testing: An added value to virological monitoring?

**DOI:** 10.1371/journal.pone.0228192

**Published:** 2020-02-05

**Authors:** Alessandra Amendola, Giuseppe Sberna, Federica Forbici, Isabella Abbate, Patrizia Lorenzini, Carmela Pinnetti, Andrea Antinori, Maria Rosaria Capobianchi

**Affiliations:** 1 Laboratory of Virology, National Institute for Infectious Diseases “Lazzaro Spallanzani” IRCCS, Rome, Italy; 2 Clinical Department, National Institute for Infectious Diseases “Lazzaro Spallanzani” IRCCS, Rome, Italy; "INSERM", FRANCE

## Abstract

New methods of HIV-1 RNA quantification based on dual-target detection are increasingly used in HIV viral load monitoring, but clinical implications and impact of dual-target detection on HIV-1 infection management are not established. Aptima HIV-1 Quant Dx assay is a last generation HIV viral load method, that uses *pol* and *LTR* as simultaneous target, providing quantitative results based mainly on *pol* target, while *LTR* target is used to report the results when *pol* signal is absent. In our laboratory, about 6% of results of all HIV-1 viral load tests performed with this platform in one year period resulted from *LTR* signal. Interestingly, *LTR*-based viremia (sometimes exceeding 1,000 copies/mL) was observed in a small proportion (up to 1%) of patients under ART, considered for long time virologically suppressed on the basis of a single target (*pol*-based) assay. Male gender, >700 *vs* <200 CD4 cell/mL and dual therapy including NRTI plus either NNRTI, or PI/b or INSTI were independently associated with increased risk of *LTR*-based HIV-1 viral load detection by multivariable logistic regression. A significant linear correlation was observed between *LTR*-based HIV-1 RNA levels and PBMC-associated proviral DNA. Moreover, in a small group of patients with HIV-1 RNA levels >200 copies/mL, longitudinal assessments showed parallel kinetics between plasma viremia and proviral DNA. Sequencing of *pol* region for drug resistance assessment in patients with *LTR*-based viremia failed on plasma HIV-1 RNA, while it was successful on proviral DNA. The detection/quantification of HIV-1 viremia based only on *LTR* signal with a dual target assay in samples resulting undetectable with the more conventional target *pol* needs accurate evaluation; unravelling the biological basis of this phenomenon, here described for the first time, is mandatory to establish relevance and implication by both pathogenetic (i.e. infectivity of *LTR*-detected viruses, reservoir turnover, immune activation, etc.) and clinical standpoint.

## Introduction

According to international guidelines for clinical management of HIV-1 disease, HIV-1 RNA viral load (VL) and CD4+ T lymphocyte (CD4) cell count are the two main laboratory parameters used in clinical management of HIV-1-infected patients, before the initiation of antiretroviral therapy (ART) and in monitoring of antiretroviral treatment [DHHS. https://aidsinfo.nih.gov/guidelines.html; WHO. https://www.who.int/hiv/pub/arv/arv-2016/en/; EACS. http://www.eacsociety.org/guidelines/eacs-guidelines/eacs-guidelines.html; 1]. The level indicating optimal virological suppression is defined as VL persistently below the limit of detection of the diagnostic assay (20–75 copies/mL HIV-1 RNA, depending on the assay used), even though 50 copies/mL is conventionally considered as an acceptable threshold for viral suppression in the routine setting.

Most available HIV-1 RNA tests are based on real-time PCR, which guarantees high sensitivity, specificity, reproducibility and good correlation in overlapping range of quantification among different assays [2–5]. The design of several available HIV-1 RNA assays is based on the detection of a single target region of HIV-1 genome, such as *pol*, *gag* or *LTR*, while in the last generation assays the design tends to shift towards the simultaneous detection of two regions of viral genome. Among the latter, Aptima HIV-1 Quant Dx Assay (Aptima), a totally automated assay based on transcription mediated amplification (TMA), utilizes multiple HIV-1 specific primers to capture two regions of the HIV-1 genome. The detection of viral genome is performed in a multiplex reaction, in which both *pol* and *LTR* regions of HIV-1 are simultaneously and independently amplified [https://www.hologic.com/sites/default/files/package-insert/AW-11853-001_003_01.pdf.]. Although the viral load calculation is based on the signal provided by the *pol* target, *LTR* target is used for the VL quantification when *pol* is not amplified.

Recently, Aptima replaced Real*T*ime HIV-1 (Real*T*ime) assay (based on single-target detection of *pol*) in the Laboratory of Virology at the “Lazzaro Spallanzani” IRCCS Hospital. In a previous study, carried out in our laboratory, a comparison between these two assays yielded about 8% of discordant samples, that were detected with Aptima but were undetected with Real*T*ime [6], in line with other report [7]. After the implementation of Aptima as routine VL test, we also observed that some patients, historically considered fully suppressed with the previous method (Real*T*ime), showed measurable HIV-1 RNA detected only with *LTR* target. For this reason we analyzed the frequency, among our patient population, of VL results based only on *LTR* detection, the reproducibility of *LTR*-based HIV-1 viremia results, and the characteristics of patients displaying *LTR*-based viremia. To identify possible factors associated with this feature. Indeed, these data may have considerable implication in clinical management of HIV-1 infection and could reveal unanticipated pathogenetic scenarios.

## Materials and methods

### Clinical samples

A total of 14,865 samples from chronically HIV-1 infected patients attending the out-patient care facility at “Lazzaro Spallanzani” Hospital in Rome for routine monitoring of HIV-1 VL, were collected from 3^th^ May 2018 to 2^nd^ May 2019. Samples were obtained from whole-blood collected in EDTA-containing tubes by centrifugation (1,100g for 20 min) directly after arrival at the laboratory and tested in same day or the subsequent day with Aptima.

The study was carried out on residual samples submitted for diagnostic purpose. Ethical clearance was obtained from the Institutional Ethical Committee (Comitato Etico dell’Istituto Nazionale per le Malattie Infettive Lazzaro Spallanzani I.R.C.C.S.), and written informed consent was deemed unnecessary, since all samples were anonymized before testing.

After routine VL measurement, all residual plasma and PBMC samples were stored at -20°C or -80°C for additional investigation. Plasma specimens quantified by Aptima with *LTR* region were used afterwards (within 1–3 months from withdrawal) to evaluate the inter-assay concordance with other HIV-1 VL assays.

Demographic and clinical characteristics of patients enrolled in this study were analyzed and described in Results.

### HIV-1 VL assays

All VL assays were performed according to the manufacturers’ instructions following product inserts [Aptima HIV-1 Quant Dx assay (Aptima) by Hologic, Inc., San Diego, CA; Real*T*ime HIV-1 (Real*T*ime) by Abbott Molecular Inc., Des Plaines, IL; COBAS1 AmpliPrep/COBAS1 TaqMan1 HIV-1 Test, v2.0 (CAP/CTM) by Roche Molecular Diagnostics Inc., Pleasanton, CA]. All assays used in this comparison automatically report VL results in copies/mL according to their respective conversion factor (as declared by the manufacturer).

Aptima was used on the fully automated Panther system. The assay requires a sample volume of 0.7 mL and reports quantitative HIV-1 results in a range of 30 to 10,000,000 copies/mL. The 6.2.3.1 software version, showing both *pol*- and *LTR*-based viremia values, was made available by the assay producer. To assess assay reproducibility of HIV-1 VL results when the calculation was based on the *LTR* signal, 100 consecutive samples falling in this category underwent same day retesting, including 40 samples with <30 copies/mL, 40 samples with 100–200 copies/L and 20 samples with >200 copies/mL.

Real*T*ime was used on the semi-automated *m*2000 system. It requires a sample volume of 0.8 mL and reports quantitative HIV-1 results in a range of 40 to 10,000,000 copies/mL. This test targets *pol* (*integrase*) region.

CAP/CTM was performed on the docked configuration of the Cobas Ampliprep and TaqMan 96 system. The assay requires at least 1.0 mL of specimen to report quantitative HIV-1 RNA results between 20 and 10,000,000 copies/mL. Like Aptima, it is based on dual-target detection of HIV (*gag* and *LTR* regions), but for individual samples it does not provide information on the target actually used for quantification.

PBMC-associated total HIV-1 DNA was quantified by in-house real time PCR, targeting *LTR* region (sensitivity: 10 copies/reaction); an *hTERT-*targeting in-house real time PCR was used to refer the HIV-1 DNA copies to 10^6^ PBMC [8].

### HIV-1 genotypic resistance testing and subtyping

HIV-1 genotyping resistance testing (GRT) was carried out on plasma samples and PBMC-associated HIV-1 DNA with ViroSeq HIV-1 Genotyping System (Abbott Molecular Inc., Des Plaines, IL), targeting the *pol* region, according to the manufacturers’ instructions.

HIV-1 subtype was established on the basis of HIV-1 *pol* sequences obtained for baseline GRT testing before ART initiation. These were aligned in BioEdit and compared to reference sequences for major HIV-1 and circulating recombinant forms (CRFs), available on the Los Alamos database [http://www.hiv.lanl.gov]. Subtype classification was confirmed using REGA [http://www.bioafrica.net/rega-genotype/html/subtypinghiv.html] and COMET [http://comet.retrovirology.lu/] subtyping tools. In case of discordant results between the tools, subtype was assigned on the base of RDP4 [http://rdp4.software.informer.com/4.1/] and of phylogenetic analysis, using MEGA6 software [http://www.megasoftware.net/].

### Statistical analysis

All data were analyzed using STATA version 15.1 and GraphPad Prism 4. Median values and interquartile ranges (IQR) were used to describe numerical variables, while counts and percentages were employed for qualitative variables. The comparisons between samples with and without detectable HIV-1 RNA in *LTR* were performed using Chi-square test for categorical variables and Mann-Whitney test for continuous measures. Pearson’s correlation analysis was used to establish the correlation between HIV-1 VL and proviral DNA levels. Two-tailed *p*-values <0.05 was considered statistically significant.

Covariates included in the analyses were: gender, HIV transmission mode (MSM, heterosexual, IDU), age, nationality (Italian *versus* non-Italian), years of HIV-1 infection, CD4 (0–350, 351–500, 501–700, >700), CD4/CD8 ratio (either <1 *versus* ≥1 or median values), HIV-1 RNA (≤50 *versus* >50 copies/mL), ART regimens. Multivariable logistic regression was used to identify factors independently associated with HIV-1 RNA detected through *LTR* signal only.

## Results

### Analysis of VL obtained with Aptima according to the HIV-1 amplified target

Although Aptima exploits dual-target approach to detect and quantify HIV-1 RNA, VL results calculated by the platform software derive from one of the two amplified targets: on a total of 14,865 plasma clinical samples, 13,972 (93.99%) were quantified with *pol* signal, while 893 (6.01%) were based only on the signal provided by *LTR*.

**Fig 1:**
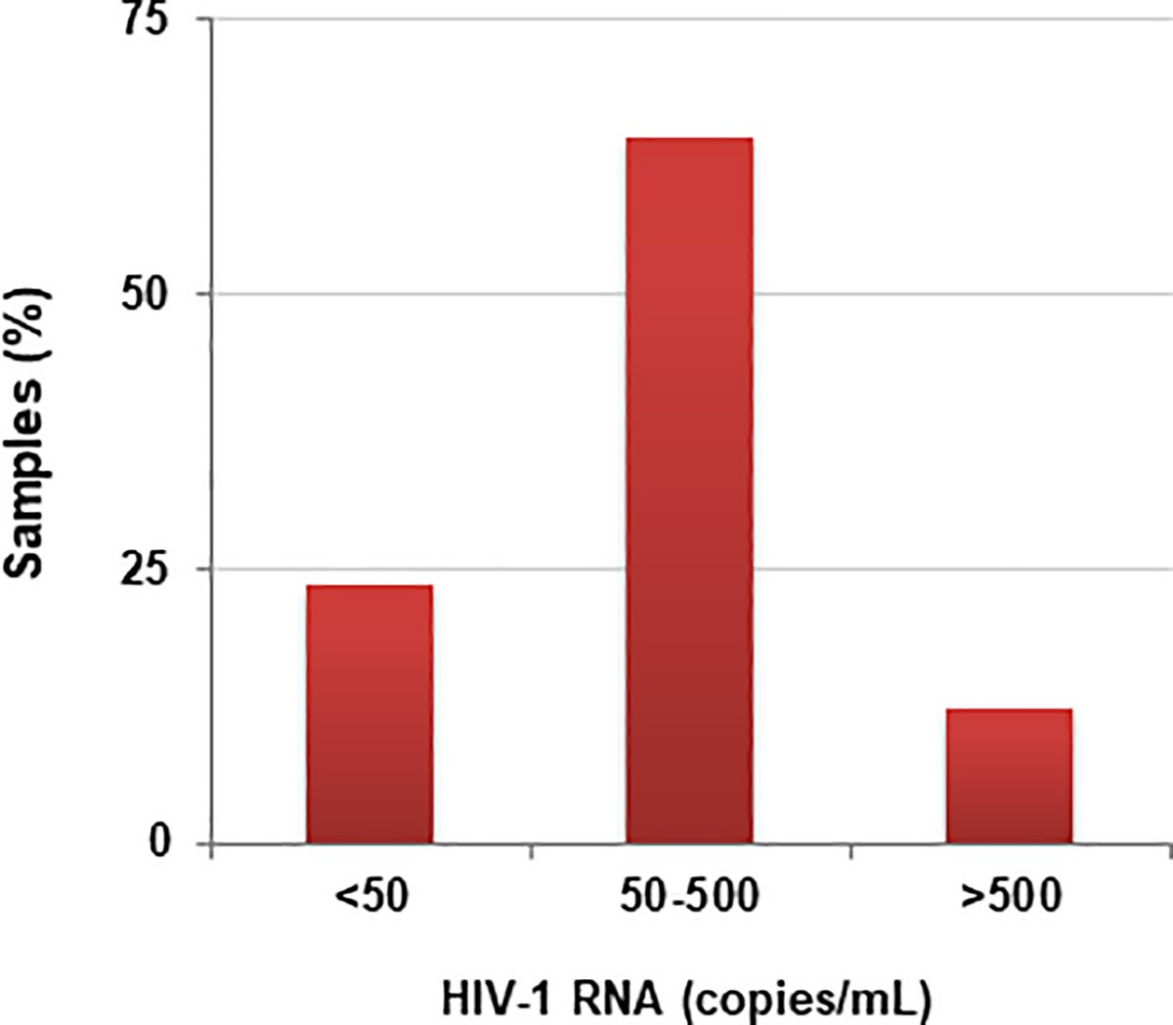
Distribution of samples (n = 148) that were quantified with the *LTR* signal according to HIV-1 RNA ranges (<50 copies/mL; 50–500 copies/mL; >500 copies/mL) by Aptima.

Among the specimens whose measurement was based only on *LTR*, 745 (5.0% of total) exhibited HIV-1 RNA detected below <30 copies/mL and 148 (1.0%) were accurately quantified (mean and min-max VL values: 355 and 30–9182 copies/mL HIV-1 RNA). Most of these samples (n = 95, 0.64% of total samples) were in the range 50–500 copies/mL, while a minority of samples (n = 19, 0.13% of total samples) displayed HIV-1 RNA values >500 copies/mL (Fig 1)

When same day retesting was performed, reproducibility of results showed a gradient, depending on the VL value: 38% of results from samples with VL <30 copies/mL HIV-1 RNA were confirmed; 80% of samples with HIV-1 RNA values in the range of 100–200 copies/mL and virtually 100% of samples with >200 copies/ml provided results consistent with the original measurement.

During the study period, 722 patients displayed VL detected only on the basis of *LTR* signal in two or more visits: most of them (n = 624; 86.43%) showed one or two VL <50 copies/mL with *LTR* region, while 69 (9,56%) had one or two VL >50 copies/mL. Eleven patients (1.52%) had three or more confirmed *LTR*-based values, and 7 of them showed at least one value of viremia >200 copies/mL (mean: 1,914 copies/mL; median: 347 copies/mL HIV-1 RNA).

**Fig 2:**
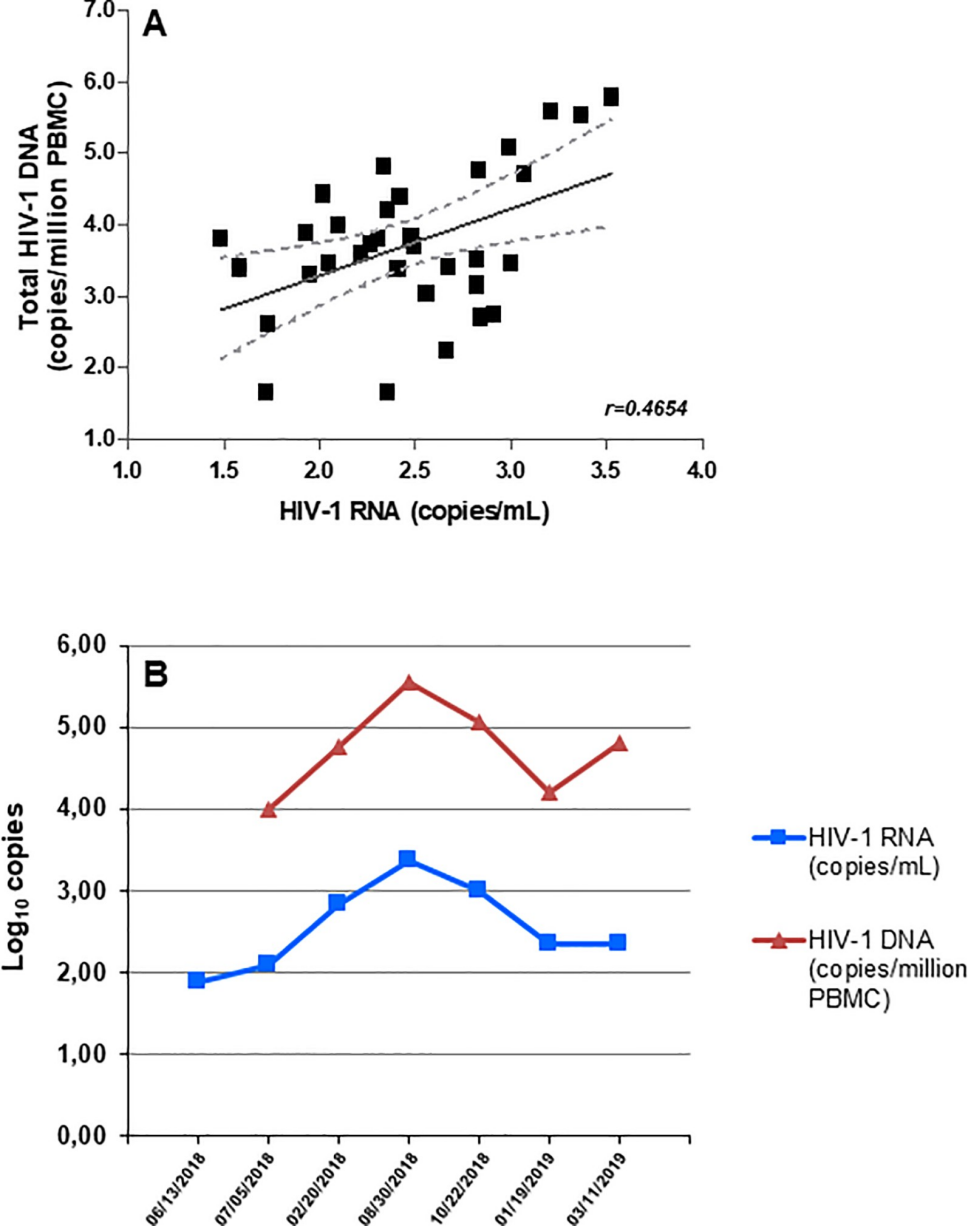
Correlation between *LTR*-detected HIV-1 RNA and PBMC-associated total HIV-1 DNA levels. (A) Correlation between VL levels detected by *LTR* region and total HIV-1 DNA content measured in PBMC in 33 samples (*p* = 0.0063; 95% confidence interval of the regression, shown with dashed lines). (B) Kinetics of plasma HIV-1 RNA (blue square symbols) and PBMC-associated total HIV-1 DNA (red triangle symbols) in one representative patient, showing viremia repeatedly detected with *LTR* target.

Linear regression analysis performed on 33 samples, for which *LTR*-based HIV-1 RNA and proviral DNA were available, indicated significant correlation (r = 0.4654, *p* = 0.0063) between the two parameters (Fig 2, Panel A). In addition, in a patient for whom multiple longitudinal evaluation had been performed, a parallel kinetics was observed for HIV-1 RNA and proviral HIV-1 DNA (Fig 2, Panel B). Parallel trend of the two virological parameters suggests a close connection between the two compartments.

### Comparison between dual-target Aptima and single-target Real*T*ime assays

Residual plasma of 114 samples with VL detected (<30 copies/mL) or quantified with *LTR* signal by Aptima were retested with Real*T*ime (LLOD: 40 copies/mL); the results are shown in Table 1. As can be seen, most (n = 91; 79.7%) of the samples detected or quantified with *LTR* signal by Aptima resulted not detected with Real*T*ime. All but one of the remaining resulted detected <40 copies/mL with Real*T*ime.

**Table 1:** Comparison of VL values obtained with Aptima (*pol* and *LTR* dual-target) and Real*T*ime (*pol* single-target) in 114 samples with viremia quantified with *LTR* region.

	Aptima (HIV-1 RNA copies/mL)		
Real*T*ime (HIV-1 RNA copies/mL)	<40^a^	>40^a^	Total
Not detected	59	32	91
<40	11	11	22
>40	0	1	1
Total	70	44	114

As Aptima and Real*T*ime show different LLOD (<30 and <40 copies/mL HIV-1 RNA, respectively), comparison of results of viremia was carried out standardizing the results to the higher LLOD (40 copies/mL) of Real*T*ime.

^**a**^Comparison was referred to LLOD of RealTime (40 copies/mL).

### Comparison of VL obtained with Aptima and other two assays in patients previously considered virologically suppressed with *pol*-based single-target procedure

**Fig 3:**
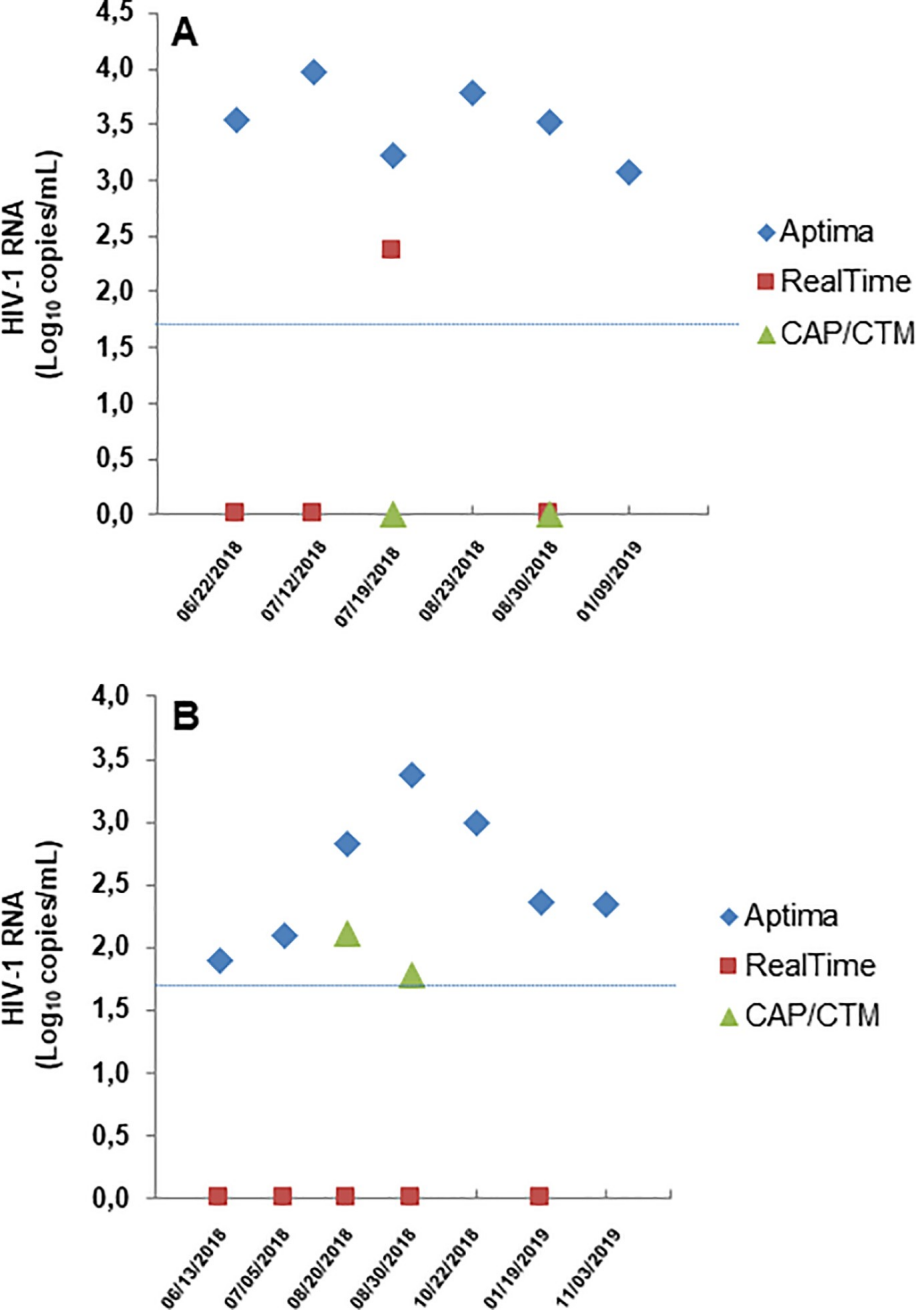
Comparison of HIV-1 VL obtained with Aptima, Real*T*ime and or CAP/CTM in longitudinal samples from two patients with HIV-RNA detected only with *LTR* signal. Residual plasma samples quantified with *LTR* region by Aptima were re-tested with Real*T*ime (n = 4 for patient A and n = 5 for patient B) and CAP/CTM (n = 2 for both patient A and B). Symbols: red square, Real*T*ime result; green triangle, CAP/CTM result; blue diamond, Amtima LTR-based result. Light blue dot line indicates the conventional suppressed viremia, i.e. 50 copies/mL HIV-1 RNA.

We selected two HIV-infected patients showing multiple VL data repeatedly measured with *LTR* signal by Aptima. According to previous viremia results obtained with Real*T*ime (steadily <40 copies/mL HIV-1 RNA), these patients had been considered virologically suppressed for 5–10 years. Residual plasma samples from these patients resulting viremic with *LTR*-quantified HIV-1 RNA underwent further assessment with Real*T*ime and/or CAP/CTM. As shown in Fig 3, in one patient (Panel A), six longitudinal Aptima tests provided HIV-1 VL results, ranging from 1,184 to 9,182 copies/mL. Some of these samples were also tested with Real*T*ime and CAP/CTM, resulting all but one (231 copies/mL), not-detected with Real*T*ime and not-detected with CAP/CTM (although *gag*- and *LTR*-based procedure). Unfortunately, it was not possible to re-test the last two samples resulted undetectable with CAP/CTM and the sample quantified by Real*T*ime for insufficient residual sample. It is worth to note that a sample collected in 2015 and resulting at that time with HIV-1 RNA undetectable with Real*T*ime, stored at -80°C and never thawed before, was re-tested with Aptima and showed 1,031 copies/mL based on *LTR* signal, confirming persistent *LTR*-based VL dated at least 4 years back the introduction of the Aptima assay in the laboratory. In another patient (Fig 3, Panel B), seven longitudinal Aptima tests provided HIV-1 VL results ranging from 78 to 2,360 copies/mL; CAP/CTM was able to quantify the viremia in 2 of such samples, while all the 5 samples retested with Real*T*ime yielded undetectable HIV-1 VL.

### Genotypic drug resistance and HIV-1 subtypes in samples with *LTR*-detected VL

GRT was not successful on HIV-1 RNA plasma samples from patients showing VL results based on *LTR* signal, due to the lack of PCR amplification of *pol* region (target region also for this genotyping test), consistent with results of Aptima and Real*T*ime assays. On the contrary, the GRT performed on the proviral DNA from five such patients was successful, revealing the presence of amplifiable *pol* sequence in at least a subpopulation of archived virus genomes.

**Fig 4:**
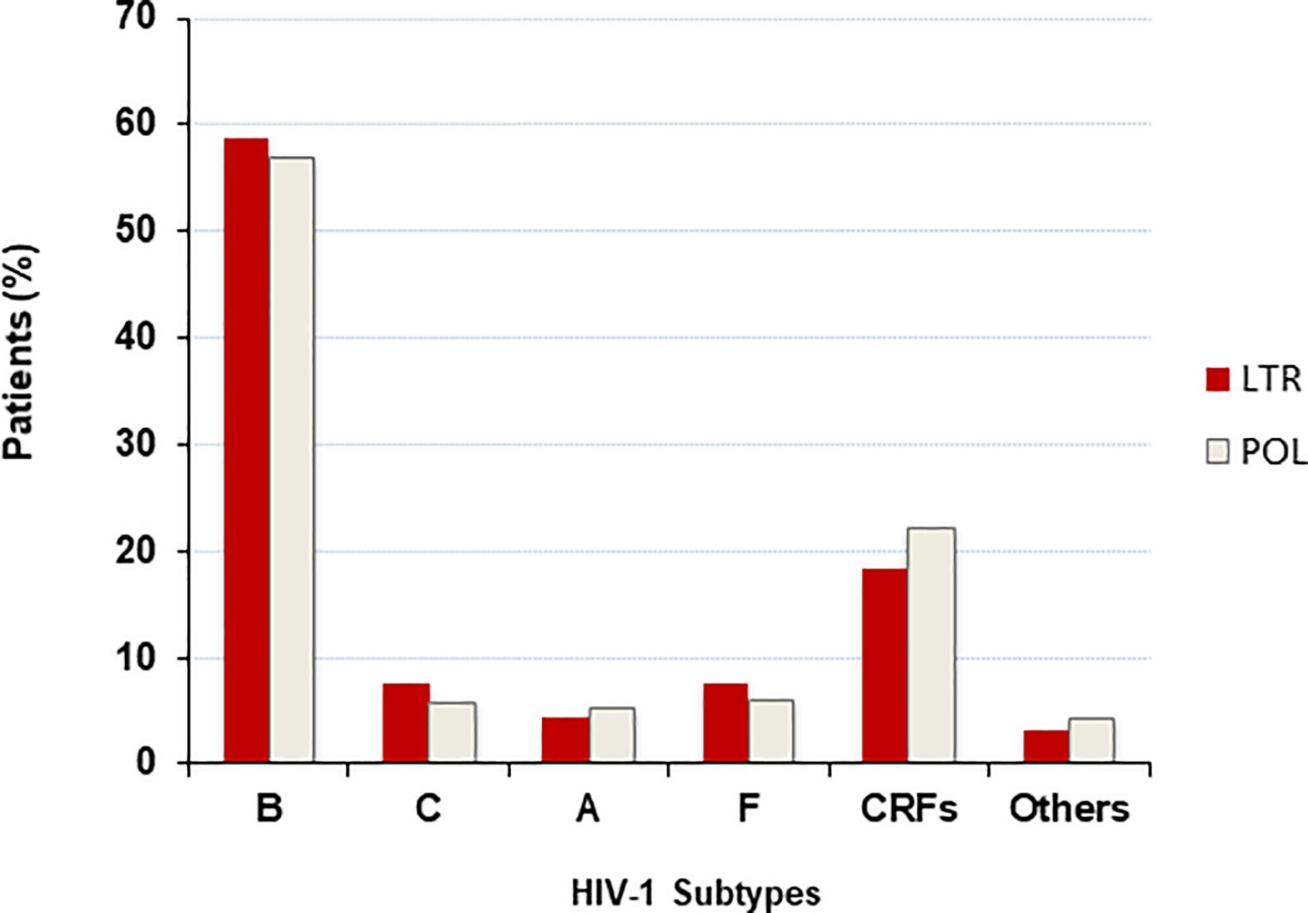
Distribution of HIV-1 subtypes in patients whose VL result was based on the *LTR* or *pol* signal. Dark red columns represent the HIV-1 subtype distribution (%) in patients whose viremia was detected with *LTR* region (n = 92); light grey columns refer to patients whose VL was calculated on the basis of *pol* signal (n = 1,749).

Subtype information was available for 92 patients whose VL was based on *LTR* signal: 58,70% were HIV-1 subtype B (3 of which were B/F), 18.47% were CRFs (n = 5 CRF12_BF; n = 1 CRF28_BF; n = 1 CRF39_BF; n = 2 CRF60_BC; n = 2 CRF31_BC; n = 1 CRF20_BG; n = 1 CRF02_AC; n = 4 CRF02_AG), 7.6% subtype C, 7.6% subtype F, 4.3% subtype A, 3.26% were J/K subtype. A similar distribution of HIV-1 subtypes was observed in the overall HIV-1 infected population referred to our center for therapeutic management, whose VL was based on *pol* target (Fig 4).

### Patients’ characteristics and factors related to HIV-1 RNA detected with *LTR*

All the patients whose VL was detected only with *LTR* were receiving ART at the sampling time. ART had been prescribed for a median of 4.6 years (IQR 1.2–10.3) and HIV-RNA detected with *LTR*-region by Aptima was <50 copies/mL in 84.5% of cases. The median CD4 and CD8 were 715 cell/mL (IQR 537–874) and 790 cell/mL (IQR 591–1044), respectively; CD4/CD8 ratio was 0.87 (IQR 0.60–1.14). A positive HCV serology was found in 17.4% of patients, HBsAg positivity in 6.5%.

To explore possible factors associated with HIV-1 VL detection based only on *LTR* signal, a comparison with patients with *pol*-based VL whose clinical information were available was performed. Due to the transient nature of some of the analyzed parameter, according to the time of VL determination, each parameter included in the analysis refers to the same date of the corresponding VL. On the whole, data from a total of 103 samples from 95 patients measured with *LTR*-based signal and 2,583 samples from 1,386 patients measured with *pol*-based signal were included in the univariate and multivariate analysis (Table 2). Males significantly (*p* = 0.028) predominate in the group of patients with VL detected only with *LTR*, compared to *pol*-detected patients. Concerning immunological data, median CD4 count and CD4/CD8 ratio were higher in samples with *LTR*-based VL (*p* = <0.001 and *p* = 0.002, respectively). At multivariable analysis male gender *vs* female, higher number of CD4 cells (>700 cell/mL *vs* <350) and dual ART based on one NRTI + either NNRTI or PI/b or INSTI (*vs* those on 2NRTI+NNRTI) remained independently associated with *LTR*-detected VL.

**Table 2:** Comparison of demographic-viro-immunological characteristics observed in patients with VL detected with *pol* or *LTR* regions by Aptima and logistic regression model of factors associated with VL detected with *LTR* region, but not with *pol*.

Characteristics	Univariable					Multivariable			
	*pol*-detected VL n = 2583		*LTR*-detected VL n = 103		*p*-value	Odds Ratio	95% CI		*p*-value
**Gender**	n	%	n	%					
Female	560	21.7	13	12.6	**0.028**	1.00			
Male	2023	78.3	90	87.4		2.38	1.22	4.62	**0.011**
**HIV-1 transmission**	n	%	n	%					
Heterosexual	801	31.0	32	31.1	0.542	1.00			
MSM	1075	41.6	46	44.7		0.70	0.41	1.20	0.194
IDU	332	13.3	10	9.7		0.71	0.32	1.58	0.402
Others	6	0.2	1	0.9		6.47	0.64	65,00	0.113
Unkwnown	369	14.3	14	13.6		0.75	0.38	1.52	0.428
**Age, median (IQR)**	49	40–56	50	41–55	0.931	0.95*	0.77	1.18	0.659
*(per 10 years increase)									
**Nationality**	n	%	n	%					
Italian	2138	82.8	84	81.6	0.771	1.00			
Migrants	436	16.9	19	18.4		1.09	0.63	1.87	0.756
Missing	9	0.3	0	0					
**Years of HIV infection, median (IQR)**	7.9	1.0–18.4	8.4	2.7–16.5	0.442	0.97^	0.84	1.13	0.697
^(per 5 years increase)									
**CD4** (cell/mL)	n	%	n	%					
0–350	479	18.7	11	10.7	**0.002**	1.00			
351–500	446	17.4	9	8.7		0.77	0.31	1.92	0.574
501–700	762	29.7	31	30.1		1.59	0.75	3.37	0.228
>700	877	34.2	52	50.5		2.35	1.11	4.95	**0.025**
**CD4 (cell/mL) median (IQR)**	594	413–773	715	537–874	**<0.001**				
**CD8 (cell/mL) median (IQR)**	770	572–1037	790	591–1044	0.422				
**CD4 to CD8 ratio**	n	%	n	%					
<1	1792	69.9	65	63.1	0.142	1.00			
≥1	772	30.1	38	36.9		1.03	0.65	1.63	0.902
**Ratio median (IQR)**	0.77	0.49–1.10	0.87	0.60–1.14	**0.031**				
**HIV-1 RNA** (copies/mL)	n	%	n	%					
≤50	2084	80.7	87	84.5	0.339	1.00			
>50	499	19.3	16	15.5		0.95	0.49	1.85	0.883
**Type of ART regimens**	n	%	n	%					
2NRTI+NNRTI	628	24.3	25	24.3	0.085	1.00			
2NRTI+PI/b	209	8.0	10	9.7		1.21	0.54	2.69	0.644
2NRTI+INSTI	743	28.8	23	22.3		0.86	0.47	1.58	0.630
Dual (1 NRTI + 1 between NNRTI or PI/b or INSTI)	375	14.5	25	24.3		1.91	1.06	3.42	**0.031**
Dual (2 among NNRTI, PI/b, INSTI)	147	5.7	7	6.8		1.68	0.69	4.10	0.254
PI/b-monotherapy	89	3.5	4	3.9		1.28	0.43	3.82	0.662
Others	392	15.2	9	8.7		0.64	0.26	1.59	0.335

## Conclusions

This study is the first report specifically addressing the detection/quantification of HIV-1 viremia based only on *LTR* signal with a dual target assay in samples resulting undetectable with the more conventional target *pol*. Aptima is a dual-target nucleic acid amplification assay for HIV-1 RNA monitoring during ART. With this assay, we observed 20.02% more samples with VL detected below 40 copies/mL and 19.77% less samples with undetected VL in comparison with the single-target based Real*T*ime assay. This finding can be explained by differences in sensitivity (12 *vs* 29 copies/mL) [https://www.hologic.com/sites/default/files/package-insert/AW-11853-001_003_01.pdf.; https://www.molecular.abbott/us/en/products/infectious-disease/realtime-hiv-1-viral-load], amplification principle (TMA *vs* real-time PCR), as well as test design: dual *vs* single-target detection.

The dichotomy between *pol*- and *LTR*-based HIV-1 RNA detection by Aptima in a group of patients, although of reduced size, may be relevant for clinical management, and opens several questions, concerning technical, therapeutic and pathogenetic aspects. The first question is the correlation between VL data obtained with systems based on single or dual-target capture of HIV-1 genome. In numerous studies, optimal correlation between Aptima and Real*T*ime [4–7], or Aptima and CAP/CTM [5, 7] have been observed when results are considered on the whole, consequently the assay results seem interchangeable. Nevertheless, it should be considered that, although quantitative results produced by Aptima are generated by default from the signal provided with *pol* amplification, *LTR* signal is used for VL measurement when *pol* target is absent: in our experience, this has been observed in 6% of samples, consistent with the previously reported rate of discordant results observed when Aptima was compared to a *pol* single-target assay [6]. This phenomenon, at least for VL values >200 copies/mL, does not appear to be attributable to a laboratory artifact, since in almost 100% of cases re-testing confirmed *LTR* amplification. In specimens with *LTR*-detected VL between 30 and 199 copies/mL the subsequent re-test confirmed the presence of the *LTR* signal in the 62.5% of samples. As expected, re-testing of samples with VL <30 copies/mL (based on *LTR* signal) confirmed this result in less than 38% of the samples, while the others resulted not-detected.

The second question concerns the effective significance of VL data obtained with *LTR* region and their implications in clinical management of patients under ART. Indeed, most guidelines and various studies strongly recommend to pay attention to patients showing ≥2 consecutive HIV-1 VL above 200 copies/mL, as this finding may predict an imminent failure of ART, virus evolution and emergence of drug resistance [DHHS. https://aidsinfo.nih.gov/guidelines.html; WHO. https://www.who.int/hiv/pub/arv/arv-2016/en/; EACS. http://www.eacsociety.org/guidelines/eacs-guidelines/eacs-guidelines.html; 1; 9–10]. Recently, also low levels of viremia between 51 and 200 copies/mL have been associated with risk of virologic failure, particularly among ART-experienced patients [11]. In this study, based on the *LTR* signal, a small number of patients showed a VL result in the range considered at risk of virological failure: almost 1% of HIV-infected patients followed at our institution showed ≥2 VL results above 50 copies/mL (in the range 50–500 copies/mL), of whom about half with at least one VL result >200 copies/mL.

Based on current management of HIV patients, the detection of VL >50copies/mL in patients previously considered virological suppressed, would trigger intensified virological monitoring in view of possible changes in the therapeutic plan. Since, to date, all diagnostic systems for HIV-1 RNA monitoring uses single/double target detection with a unique undifferentiated amplification signal, none of the guidelines has ever taken into consideration the possible differences deriving from different viral genome targets, nor the impact of using double or even multiple target regions.

Now, with the new Aptima assay based on double-target and double differentiated signal for HIV-1 RNA detection, a more complex VL result is generated, which contains additional information as compared to the previous methods, i.e. the target region used to calculate plasma viremia. It is mandatory to address the biological significance of this more complex VL result, to appropriately use this information in the clinical setting. The *pol* and *gag* regions of HIV-1 genome are conserved genomic regions, but are also primary targets for ART; hence, these regions are subject to strong selective pressure that could favor the appearance of genomic alterations responsible for the non-detectability of VL. On the other hand, also *LTR* sequence is strongly conserved [12], since it plays a fundamental role in HIV-1 replication, expression and silencing, being the initiation site of viral transcription. Therefore, VLs detected only on *LTR* signal in patients considered virologically suppressed with *pol*- or *gag-*based assays could not only reveal the presence of alterations at level of *pol* or *gag* regions, but could also anticipate the risk of therapy failure, especially in cases where *LTR*-detected HIV-1 RNA is repeatedly confirmed in consecutive samples and shows parallel kinetics with PBMC-associated proviral HIV-1 DNA. This situation, although observed in a small number of patients, may represent a previously unrecognized clinical condition, patient-specific, that can consequently induce intensification of virological monitoring and timely assessment of drug resistance appearance. It is also possible that the isolated *LTR* signal reflects specific pathogenetic mechanisms that, in turn, may deserve a targeted therapeutic approach.

The third question concerns the reason of increased size of HIV-1 DNA reservoir observed in some patients with repeated *LTR*-detected viremia. In this phase of the study, it was not possible to analyze whether this rise depends on clonal expansion of HIV-infected cells or new cycles of viral infection. This point will be addressed in subsequent studies.

The fourth question, still connected to the increase of proviral DNA in PBMC, concerns the nature of viral particles detected through the *LTR* target, in absence of HIV *pol* signal. It is known that defective viral particles are present in the circulation of chronically infected individuals [13] and that defective HIV-1 proviruses accumulate rapidly, already during the first weeks of the infection [14]. In addition, the presence of incomplete forms of proviruses encoding translationally competent HIV RNA transcripts in HIV-infected patients under ART has been also described [15]. Therefore, the detection of VL based on *LTR* signal may reflect transcriptional activity of a defective HIV-1 *reservoir* able to produce incomplete viral particles. If this hypothesis would be proven, the VL detected with *LTR* signal could allow the recognition of such as particular patients, virologically suppressed under ART, in which both wild-type viruses suppressed by ART and defective particles revealed by *LTR* region coexist: in this cases resistance testing and therapeutic regimen switching would not be necessary.

Deep viral characterization of *LTR*-quantified VL is therefore required and, hopefully, may contribute not only to a better understanding of persistence or occasional appearance of residual viremia during efficient ART, but also to shed light on chronic immune activation status in virologically suppressed patients.

This study has some limitations. Firstly, despite the large number of clinical samples analysed, the size of the *LTR*-detected HIV-1-infected population is limited, with a prevalence of up to 1% of ART receiving patients. In addition, no similar report is available from other patient populations, so the generalizability of our findings needs to be established. Hence, our findings need to be confirmed in larger clinical studies. Secondly, this study lacks of information on patient medication adherence and concurrent immunological data.

In conclusion, the discrepancy in HIV-1 VL results derived from different target, simultaneously addressed by this modern dual target assay, needs accurate evaluation; unravelling the biological basis of this phenomenon, here described for the first time, is mandatory to establish relevance and implication by both pathogenetic (i.e. infectivity of *LTR*-detected viruses, reservoir turnover, immune activation, etc.) and clinical standpoint.

## Abbreviations

Aptima: Aptima HIV-1 Quant Dx assay
Real*T*ime: Real*T*ime HIV-1 assay
ART: antiretroviral therapy
VL: viral load
PBMC: peripheral blood mononuclear cells
LLOD: low limit of detection
IQR: Interquartile range
NRTI: nucleoside reverse transcriptase inhibitors
NNRTI: non-nucleoside reverse transcriptase inhibitors
INSTI: Integrase strand transfer inhibitors
PI/b: Protease inhibitor/boosted
