# Correction: The dual-target approach in viral HIV-1 viremia testing: An added value to virological monitoring?

**DOI:** 10.1371/journal.pone.0230018

**Published:** 2020-02-27

**Authors:** Alessandra Amendola, Giuseppe Sberna, Federica Forbici, Isabella Abbate, Patrizia Lorenzini, Carmela Pinnetti, Andrea Antinori, Maria Rosaria Capobianchi

The word “viral” incorrectly appears in the article title. The correct title is: The dual-target approach in HIV-1 viremia testing: An added value to virological monitoring? The correct citation is: Amendola A, Sberna G, Forbici F, Abbate I, Lorenzini P, Pinnetti C, et al. (2020) The dual-target approach in HIV-1 viremia testing: An added value to virological monitoring? PLoS ONE 15(2): e0228192. https://doi.org/10.1371/journal.pone.0228192
